# Outcomes of patients with chronic plaque psoriasis and hidradenitis suppurativa on biologic therapy during the Covid‐19 pandemic: A UK dermatology tertiary centre experience

**DOI:** 10.1002/ski2.54

**Published:** 2021-06-09

**Authors:** S. Khan, D. Karponis, G. Wali

**Affiliations:** ^1^ Department of Dermatology Oxford University Hospitals NHS Foundation Trust Oxford UK

**Keywords:** Hidradenitis suppurativa, medical dermatology, psoriasis, virology

## Abstract

Retrospective review data from 183 patients with psoriasis and/or hidradenitis suppurativa from a UK tertiary dermatology centre, suggests biologic therapy does not confer a significant increased risk of contracting severe Covid‐19 in this cohort. This is in line with a growing body of evidence which indicates that it is safe to continue using biologic therapies during the pandemic.

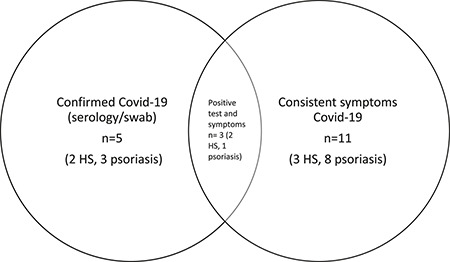

1

Dear Editor,

There has been ongoing debate about the safety of biologic therapy to treat skin disease during the Covid‐19 (SARS‐CoV‐2) virus pandemic.^1^ Recent emerging data suggests targeted systemic therapy may be associated with fewer adverse outcomes compared to no systemic therapy, although real world evidence remains limited.^2^ Poorer outcomes of Covid‐19 infection are associated with co‐morbidities such as obesity,^3^ which are more prevalent in patients with hidradenitis suppurativa (HS)^4^ and psoriasis. Furthermore, psoriasis has been suggested as a separate risk factor for death.^3^ We, therefore, sought to explore how patients with HS and psoriasis on biologic therapies have been affected.

A retrospective review of patients with psoriasis and/or HS treated with biologic therapy at our centre between January 2020 and November 2020 was performed. A total of *n* = 209 patients were identified. A total of 26 patients who were not on biologic therapy at the start of this period, or who had re‐located, were excluded leaving 164 psoriasis and 19 HS patients. We identified patients with confirmed infection (via swabs/serological testing), those with symptoms consistent with Covid‐19, those with co‐morbidities and those who shielded. Data were obtained by accessing electronic medical records, speaking with patients at routine visits/via telephone calls and is summarised in Table 1 and Figure 1.

**TABLE 1:** Characteristics of patients being treated with biological therapy

	Total cohort	Psoriasis	Hidradenitis suppurativa
No. of patients, *n* (%)	183	164 (89.6)	19 (10.4)
Male, *n* (%)	103 (56.3)	99 (60.4)	4 (21.1)
Female, *n* (%)	80 (43.7)	65 (39.6)	15 (78.9)
Mean age, years	47.5	50	42.3
Ethnicity, *n* (%)			
White	157 (85.8)	143 (87.2)	14 (73.7)
Non‐white	26 (14.2)	21 (12.8)	5 (26.3)
Biologic type, *n* (%)			
TNF alpha inhibitor	75 (41)	56 (34.1)	19 (100)
IL 17 inhibitor	21 (11.5)	21 (12.8)	0
IL 23 inhibitor	7 (3.8)	7 (4.3)	0
IL 12/23 inhibitor	80 (43.7)	80 (48.8)	0
Additional systemic therapy, *n* (%)			
Methotrexate	22 (12.0)	22 (13.4)	0
Leflunamide	2 (1.1)	2 (1.2)	0
Acitretin	1 (0.5)	1 (0.6)	0
Co‐morbidities, *n* (%)			
Any listed co‐morbidity	115 (63.8)	104 (63.4)	11 (57.9)
Psoriatic arthritis	27 (14.8)	27 (16.5)	0
Hypertension	36 (19.7)	33 (20.1)	3 (15.8)
Hypercholesterolaemia	10 (5.5)	9 (5.5)	1 (5.3)
Diabetes mellitus	21 (11.5)	20 (12.2)	1 (5.3)
Obesity	12 (6.6)	11 (6.7)	1 (5.3)
Cardiomyopathy	1 (0.5)	0	1 (5.3)
Obstructive sleep apnoea	4 (2.2)	3 (1.8)	1 (5.3)
Crohn’s disease	4 (2.2)	1 (0.6)	3 (15.8)
Covid‐19 positive (swab/serological testing), *n* (%)			
No. of patients	5 (4.8)	3 (2.9)	2 (1.9)
Symptoms	3 (2.9)	1 (0.95)	2 (1.9)
Relevant co‐morbidity (hypertension, obesity, obstructive sleep apnoea)	4 (3.8)	3 (2.9)	1 (0.95)
Gender	2 F, 3 M	1 F, 2 M	1 F, 1 M
Ethnicity	3 White, 2 Asian	2 White, 1 Asian	1 White, 1 Asian
Hospital admission	1 (0.95)	0	1 (0.95)
TNF alpha inhibitor	4 (3.8)	2 (1.9)	2 (1.9)
IL 17 inhibitor	1 (0.95)	1 (0.95)	0
Shielding at time of infection	1 (0.95)	1 (0.95)	0

**FIGURE 1:**
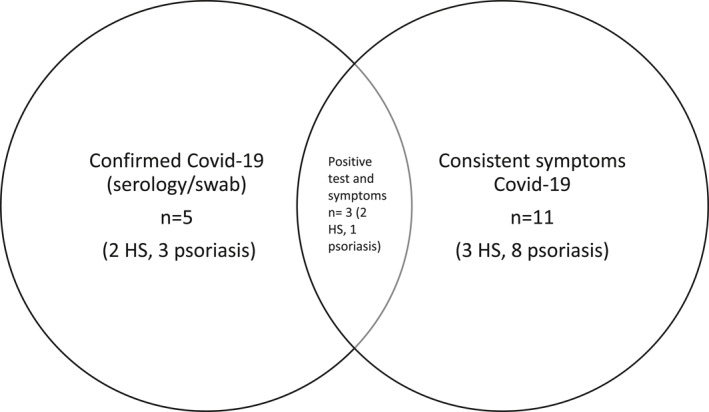
Covid‐19 symptom/testing data summary

Risk mitigating behaviour data was obtainable for 133 patients. 47%(62) shielded and 53%(71) adhered to strict social distancing. 80.5% (107) took precautions which were in line with the British Association of Dermatologists (BAD)^5^ published guidance. 7.6% (8) temporarily stopped their biologic at the start of the pandemic for fear of contracting Covid‐19 whilst being immunosuppressed.

Covid‐19 symptom/testing data was obtainable for 105 patients. Of these patients, the total number with confirmed/suspected Covid‐19 was 13 (10 psoriasis and 3 HS), 12.4%. 10/13 were not shielding at the time of infection, and 2/13 should have been as per BAD guidance.^5^ 11/13 (8 psoriasis, 3 HS) had symptoms consistent with Covid‐19 and of these, 3 tested positive (1 psoriasis, 2 HS). Two asymptomatic patients with psoriasis also tested positive, resulting in the total number of positives *n* = 5 (4.8%). Only one patient with HS required hospital admission.

In the Covid‐19 confirmed/suspected group of psoriasis patients *n* = 10, the median age was 47 years and similar to a median age of 50 in PsoProtect data.^2^ In their larger cohort of 374 confirmed/suspected Covid‐19 patients, 61% were male compared to 40% in this review. Furthermore, no patients with psoriasis were hospitalised in our cohort, compared with a rate of 21%.^2^ All of our patients recovered well supporting reports of 93% of those infected with Covid‐19 making a full recovery.^2^ There were also no deaths reported in our cohort compared to 2% in the larger study.^2^

In the Covid‐19 confirmed/suspected group of HS patients *n* = 3, the median age was 33 and 67% were female. In a study of 39 HS patients with confirmed Covid‐19,^6^ eight were admitted and one died. The majority of patients in that study were not on systemic therapy. Only one was on an anti‐TNF inhibitor (Infliximab) and had mild infection. One 55‐year‐old white male in our cohort with HS on an anti‐TNF inhibitor (Imraldi) was admitted to intensive care. He had multiple co‐morbidities (hypertension, obesity and obstructive sleep apnoea); but did not require intubation and made a good recovery.

In the confirmed positive group of psoriasis and HS patients *n* = 5, 4/5 were on anti‐TNF inhibitors, which is unsurprising as 41% of the total cohort were on this type of biologic. 1/5 was on an IL‐17 inhibitor. Interestingly, PsoProtect data^2^ showed a higher frequency of hospitalisation amongst psoriasis patients on IL‐23 inhibitors compared with anti‐TNF and IL‐17 inhibitors; however, this was not found to be statistically significant, thus warranting larger studies to establish any possible association.

We are mindful of our small sample size and whilst definite conclusions cannot be drawn, overall our data adds to the growing body of evidence^2^, ^7^ that biologic therapy does not confer a significant increased risk of contracting severe Covid‐19 in patients with psoriasis/HS, which suggests that it is safe to continue using biologic therapies during the pandemic. Whilst we did not compare outcomes of patients who had psoriasis/HS on biologics to those on other systemics only, out of 25 (15.2%) psoriasis patients on additional systemic medications, none tested positive for Covid‐19 infection. Shielding behaviour is likely to have contributed to the lower risk of adverse Covid‐19 outcomes and is likely to be an important potential mediator in these groups of patients as suggested by the PsoProtect and CORE‐UK study groups.^8^

## CONFLICTS OF INTEREST

None to declare.

## References

[1] Price KN , Frew JW , Hsiao JL , Shi VY . COVID‐19 and immunomodulator/immunosuppressant use in dermatology. J Am Acad Dermatol. 2020;82 (5):e173–e175. May. 32224277 10.1016/j.jaad.2020.03.046 PMC7156805

[2] Mahil SK , Dand N , Mason KJ , Yiu ZZN , Tsakok T , Meynell F , et al. Factors associated with adverse COVID‐19 outcomes in patients with psoriasis – insights from a global registry‐based study. J Allergy Clin Immunol. 2020. 10.1016/j.jaci.2020.10.007 PMC7566694 33075408

[3] Williamson EJ , Walker AJ , Bhaskaran K , Bacon S , Bates C , Morton CE , et al. OpenSAFELY: factors associated with COVID‐19 death in 17 million patients. Nature. 2020;584:1–436. 10.1038/s41586-020-2521-4 PMC7611074 32640463

[4] Seltzer JA , Okeke CAV , Perry JD , Shipman WD , Okoye GA , Byrd AS . Exploring the risk of severe COVID‐19 infection in patients with hidradenitis suppurativa. J Am Acad Dermatol. 2020;83:e153–e154. 32389715 10.1016/j.jaad.2020.05.012 PMC7205672

[5] British Association of Dermatologists . Dermatology Advice Regarding Medication Acting on the Immune System: Adults, Paediatrics and Young People. Available online. https://www.bad.org.uk/shared/get‐file.ashx?itemtype=document&id=6674

[6] Rozzo G , Ramondetta A , Fierro MT , Dapavo P , Ribero S . Moderate‐to‐severe hidradenitis suppurativa under systemic therapy during the COVID‐19 outbreak. Dermatol Ther. 2020;33. 10.1111/dth.13680 PMC7283708 32447800

[7] Cho SI , Kim YE , Jo SJ . Association of COVID‐19 with skin diseases and relevant biologics: a cross‐sectional study using nationwide claim data in South Korea. Br J Dermatol. 2020 Sep 2;184:296–303. 10.1111/bjd.19507 32875557 PMC9213995

[8] Mahil SK , Yates M , Langan SM , Yiu ZZN , Tsakok T , Dand N , et al. Risk mitigating behaviours in people with inflammatory joint and skin disease during the COVID‐19 pandemic differ by treatment type: a cross‐sectional patient survey. Br J Dermatol. 2020 Dec 23. 10.1111/bjd.19755 PMC9214088 33368145

